# *EHP* Paper of the Year, 2012

**DOI:** 10.1289/ehp.1206266

**Published:** 2012-12-03

**Authors:** Hugh A. Tilson

**Affiliations:** Editor-in-Chief, *EHP*, E-mail: tilsonha@niehs.nih.gov

*Environmental Health Perspectives* (*EHP*) established the Paper of the Year Award in 2008 to recognize high-quality papers published in the journal (Tilson 2008). Starting in 2011, *EHP* decided to recognize two papers each year (Tilson 2011). The *EHP* Classic Paper of the Year is the research article, commentary, or review that is the most highly cited over the preceding 60 months. The winner of the 2012 *EHP* Classic Paper of the Year, announced last July, was “Exposure of the U.S. Population to Bisphenol A and 4-*tertiary*-Octylphenol: 2003–2004” by Antonia M. Calafat, Xiaoyun Ye, Lee-Yang Wong, John A. Reidy, and the late Larry L. Needham of the Division of Laboratory Sciences, National Center for Environmental Health, Centers for Disease Control and Prevention (Tilson 2012). The second paper to be recognized each year is the *EHP* Paper of the Year. This award recognizes the most highly cited paper published during the preceding year. Both awards are subject to the approval of the *EHP* Board of Associate Editors.

In this issue, *EHP* is pleased to announce that the 2012 Paper of the Year award will be shared by three papers published in the same issue: “Prenatal Exposure to Organophosphates, Paraoxonase 1, and Cognitive Development in Childhood” by Stephanie M. Engel, James Wetmur, Jia Chen, Chenbo Zhu, Dana Boyd Barr, Richard L. Canfield, and Mary S. Wolff (Engel et al. 2011); “Prenatal Exposure to Organophosphate Pesticides and IQ in 7-Year-Old Children” by Maryse F. Bouchard, Jonathan Chevrier, Kim G. Harley, Katherine Kogut, Michelle Vedar, Norma Calderon, Celina Trujillo, Caroline Johnson, Asa Bradman, Dana Boyd Barr, and Brenda Eskenazi (Bouchard et al. 2011); and “Seven-Year Neurodevelopmental Scores and Prenatal Exposure to Chlorpyrifos, a Common Agricultural Pesticide” by Virginia Rauh, Srikesh Arunajadai, Megan Horton, Frederica Perera, Lori Hoepner, Dana B. Barr, and Robin Whyatt (Rauh et al. 2011). The three studies were conducted at the Mount Sinai School of Medicine (Engel et al. 2011); the University of California, Berkeley, School of Public Health (Bouchard et al. 2011); and the Mailman School of Public Health at Columbia University (Rauh et al. 2011). The Berkeley and Mount Sinai investigators measured organophosphate (OP) pesticide metabolites in pregnant women’s urine, and the Columbia investigators measured the OP pesticide chlorpyrifos in umbilical cord blood. Children of these mothers were administered intelligence tests when they were 6–9 years of age at Mount Sinai and at 7 years of age at Berkeley and Columbia. All three studies reported associations between prenatal OP pesticide exposures and adverse effects on cognitive function that continued into early childhood. The fact that three research groups reached such similar conclusions independently adds considerable support to the validity of the findings.

*EHP* congratulates all the authors of these papers for their contribution to the environmental health science literature. These findings underscore the need to continue monitoring levels of exposure to pesticides in vulnerable populations and to study the under-lying biological changes associated with alterations of cognitive function following developmental exposure to environmental chemicals.
